# Dr. Oliver Wendell Holmes' Toast at the Dinner of the New York Odontological Society

**Published:** 1886-02

**Authors:** 


DR. OLIVER WENDELL HOLMES TOAST AT THE DINNER OF THE NEW YORK ODONTOLOGICAL SOCIETY—In declining an invitation to dine with the New York Odontological Society last week Oliver Wendell Holmes wrote: "I often think of the forlorn condition of some of the great personages of history in the days when there were no dentists, or none who would be recognized as such by the dental artists of to-day. Think of poor King David, a worn-out man at 70, probably without teeth, and certainly without spectacles. Think of poor George Washington, his teeth always ready to drop like a portcullis, and cut a sentence in two. See him in Stuart's admirable portrait, his thoughts evidently divided between the cares of empire and the maintenance of the status quo of his terrific dental arrangements. Think of Walter Savage Landor's melancholy complaint that he did not mind losing his intellectual faculties, but the loss of his teeth he felt to be a very great calamity.

"I will venture to propose, then, the dental profession and their association as its worthy representative: It has established and prolonged the reign of beauty; it has added to the charms of social intercourse and lent perfection to the accents of eloquence; it has taken from old age its most unwelcome feature and has lengthened enjoyable human life far beyond the limit of years when the toothless and purblind patriarch might well exclaim: 'I have no pleasure in them.'"

